# Recent developments in deep learning applied to protein structure prediction

**DOI:** 10.1002/prot.25824

**Published:** 2019-10-14

**Authors:** Shaun M. Kandathil, Joe G. Greener, David T. Jones

**Affiliations:** ^1^ Department of Computer Science University College London London UK; ^2^ Biomedical Data Science Laboratory The Francis Crick Institute London UK

**Keywords:** deep learning, protein structure prediction

## Abstract

Although many structural bioinformatics tools have been using neural network models for a long time, deep neural network (DNN) models have attracted considerable interest in recent years. Methods employing DNNs have had a significant impact in recent CASP experiments, notably in CASP12 and especially CASP13. In this article, we offer a brief introduction to some of the key principles and properties of DNN models and discuss why they are naturally suited to certain problems in structural bioinformatics. We also briefly discuss methodological improvements that have enabled these successes. Using the contact prediction task as an example, we also speculate why DNN models are able to produce reasonably accurate predictions even in the absence of many homologues for a given target sequence, a result that can at first glance appear surprising given the lack of input information. We end on some thoughts about how and why these types of models can be so effective, as well as a discussion on potential pitfalls.

## 1 INTRODUCTION

The field of structural bioinformatics has been using machine learning methods, and specifically artificial neural network (NN) models, for a long time. Prominent examples of early NN methods that are still widely used today are PHD,^1^, ^2^ PSIPRED,^3^ and JPred.^4^ The field has recently seen a surge of interest relating to the use of deep neural network (DNN) models. DNN models have shown excellent performance in image and language based problems, to name a few.^5^ Very recently, this excellent performance has been seen to extend to some specific CASP areas. The first application area where DNNs have had a major impact on CASP was arguably residue‐residue contact prediction, which saw a particularly marked improvement in accuracy in CASP12 and 13. In CASP13, a few groups extended these techniques further to the prediction of interatomic distances, which in some cases could then be used directly for accurate tertiary structure generation.^6^, ^7^, ^8^, ^9^, ^10^ Although not currently an area of direct interest in CASP, deep learning is also starting to show a lot of promise in the area of protein design.^11^, ^12^

This article is not intended to be a detailed exposition of every key deep learning concept; our aim is instead to provide CASP participants and observers with a working understanding of the most important DNN architectures that have been successfully applied to the core problem areas in recent CASP experiments. We will then discuss what advantages such models may have over those more traditionally used in various areas. We will then end on some thoughts on why and how these models work, their limitations, potential pitfalls, and their correct application. All discussion will be limited to supervised learning models,^13^ as the most performant DNN models used in CASP so far have been of this type.

## 2 DEEP NEURAL NETWORKS

Artificial neural networks have proven to be valuable in data modeling as they are known to be universal function approximators. This means that when configured and trained correctly, they can approximate any arbitrary continuous function to any desired approximation accuracy.^14^, ^15^ In fact, the first universal approximation theorems were proved for NN comprised of just a single hidden layer (although the theory allowed for arbitrarily many hidden units in that layer). The theorems say nothing, however, about how one might discover the network parameters that achieve a particular level of approximation, and finding good parameters for a given model architecture is achieved by the process of training. To train a NN in a supervised fashion, besides the model itself, one requires a set of training examples (a paired collection of inputs and corresponding outputs), and a cost or loss function that measures how far away from the “correct” answer a given model is. Training a NN is achieved by random initialization of the network parameters, followed by an iterative process comprising: (a) a forward pass of the NN with the current parameters to arrive at its predicted output for a training example; (b) calculation of the loss or cost for the example in question; (c) backpropagation of the loss to determine its gradient with respect to each network parameter; and (d) updating of the network parameters in proportion to the gradients. The backpropagation algorithm was popularized by a seminal paper by Rumelhart et al^16^ although the underlying ideas are much older.^17^

In general, having more artificial neurons in a model, organized in multiple layers, provides a model with a large number of adjustable parameters, and allows the model to express ever more complex functions. DNNs are, as the name suggests, composed of many layers of artificial neurons. There appears to be no consensus for how many hidden layers a network needs to have before it can be termed “deep”;^17^ a rule of thumb is that two or more hidden layers is sufficient. Of course, practical DNN models usually have many more than two hidden layers. Although an effective procedure for training these multilayer networks was developed quite early on,^18^ DNNs were rarely used in practice due to difficulties in training them; training a network of more than two hidden layers with the conventional sigmoid activation function frequently suffers from the so‐called vanishing gradient problem. Hochreiter et al^19^ describe this problem in the context of recurrent architectures, but the underlying problem is the same for deep feedforward architectures: as training the network parameters depends on the gradient of the loss function with respect to these parameters, the gradients in the earlier (nearer to the input) layers is the product of the gradients of all intermediate activations leading up to the output. This means that for small or large intermediate activation values, the resultant gradient in early layers can *vanish* (approach zero) or even sometimes *explode* (approach infinity) if the network weights are not properly tuned.

An early solution to this problem was proposed by Hinton,^20^ where deep networks were trained layer‐by‐layer using a mixture of supervised and unsupervised learning. Ultimately, this difficulty was addressed more easily by a series of seminal works that introduced new activation functions such as rectified linear units (ReLU),^21^, ^22^ new weight initialization schemes,^23^ and other innovations such as batch normalization^24^ and residual architectures^25^ to better enable the training and use of increasingly DNN models. These advances occurred side‐by‐side with advances in computing hardware, specifically the availability of affordable, fast graphics processing units (GPUs), which can also perform the mathematical operations used by NN in a massively parallel fashion.^26^, ^27^, ^28^ Many if not all of these advances are accessible via a number of freely available programming frameworks and libraries, which greatly accelerate and streamline deep learning application development. Prominent examples are Theano,^29^ mxNet,^30^ Caffe,^31^ TensorFlow,^32^ Keras,^33^ Lasagne,^34^ Torch,^35^ and PyTorch.^36^ Most of these frameworks also implement reverse‐mode automatic differentiation,^37^ a feature that hugely accelerates the application development cycle. The training of NN models by the backpropagation algorithm requires the calculation of the derivative of the loss function with respect to each parameter in each layer, and this is managed automatically by the automatic differentiation framework, using the same declarations used to build the NN model in a program. Thus, there is no need to rewrite the expressions for both the forward and reverse pass of the network, as the latter is computed from the former. This allows one to quickly experiment with different architectures for a model. It has reached the point where, within reason, as long as the NN architecture can be expressed in code (Python most usually), the network can be simulated and trained.

## 3 CONVOLUTIONAL NEURAL NETWORK (CNN) MODELS

**Figure 1:**
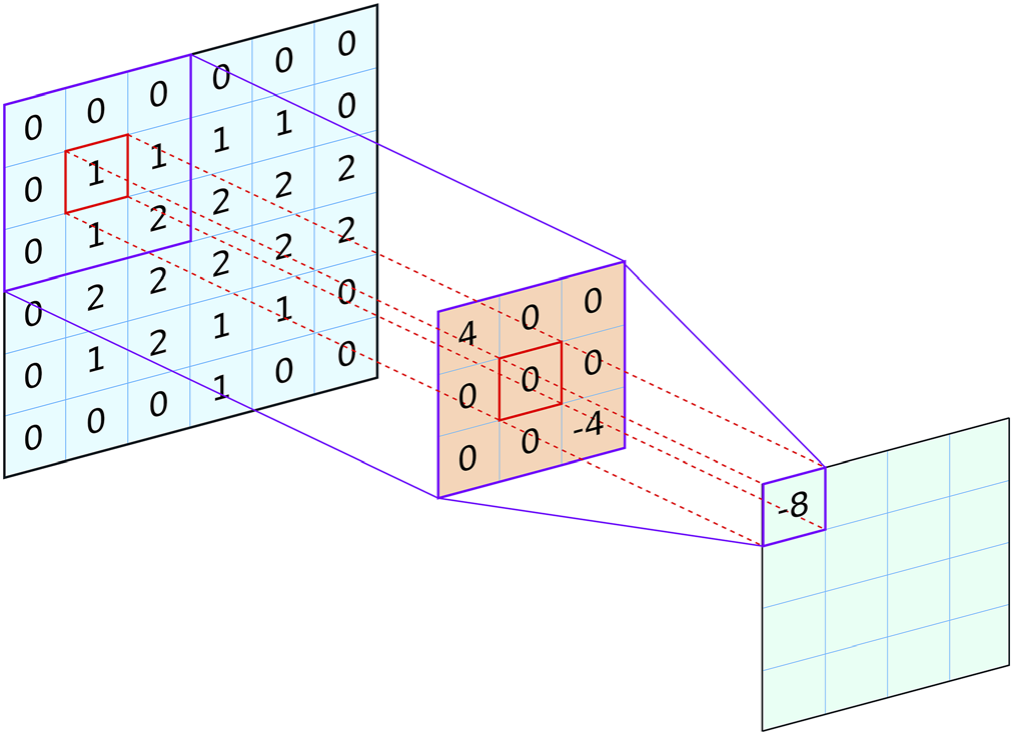
A 2D convolutional filter (orange) is applied to an input layer (blue) to obtain the values for an output layer (green). The output value (−8 in this example) is the sum of the pointwise products of the filter weights and the corresponding elements in the input (the bias is zero in this example and no nonlinear activation function is used). The same set of filter weights is used to generate the output values at every placement of the filter on the input

In the most basic implementation of a NN, all layers of artificial neurons are *fully connected*, that is, the output of any neuron in a prior layer is fed to the input of every neuron in the next layer. Convolutional nets act on 2D image‐like inputs (but can also be applied to 1D and 3D data) by applying small *filters* or *kernels* to colocated groups of pixels in the image (see Figure 1). Each filter can actually be thought of as a small single‐layer NN (perceptron), where the values in the filter are trainable weights. Although the filter is applied to every pixel in the input, the weights are shared across the whole image, and so it is equivalent to defining one single NN and applying it at every row and column position. Functionally, there is no difference between doing this and using a single sliding‐window approach, but there are clear efficiency gains from the use of convolution as it allows massive parallelism. In CASP11 and CASP12, for example, MetaPSICOV^38^, ^39^ made use of two NNs, each of which used a shallow fully connected NN and a traditional sliding‐window approach to produce competitive performance in contact prediction. With the exception that convolutional filters typically use just a single hidden layer, algorithmically the sliding window approach in MetaPSICOV is equivalent to a convolutional operation, just far less computationally efficient.

After training, the outputs from a convolutional layer operating on image‐like inputs are also image‐like, but now convey extra information, for example, the presence of an edge between two objects in the image. Convolutional architectures are suited to data that exhibit some form of spatial structure, such as images or covariance matrices. The filter weights are the same for each output pixel, meaning the network can recognize local features regardless of their spatial location in the input. As the same filter is moved across the input image to generate the output, fewer adjustable parameters are needed (as compared to a fully connected layer). Multiple filters (channels) can be learned in a single convolutional layer, each recognizing a different pattern within the data.

**Figure 2:**
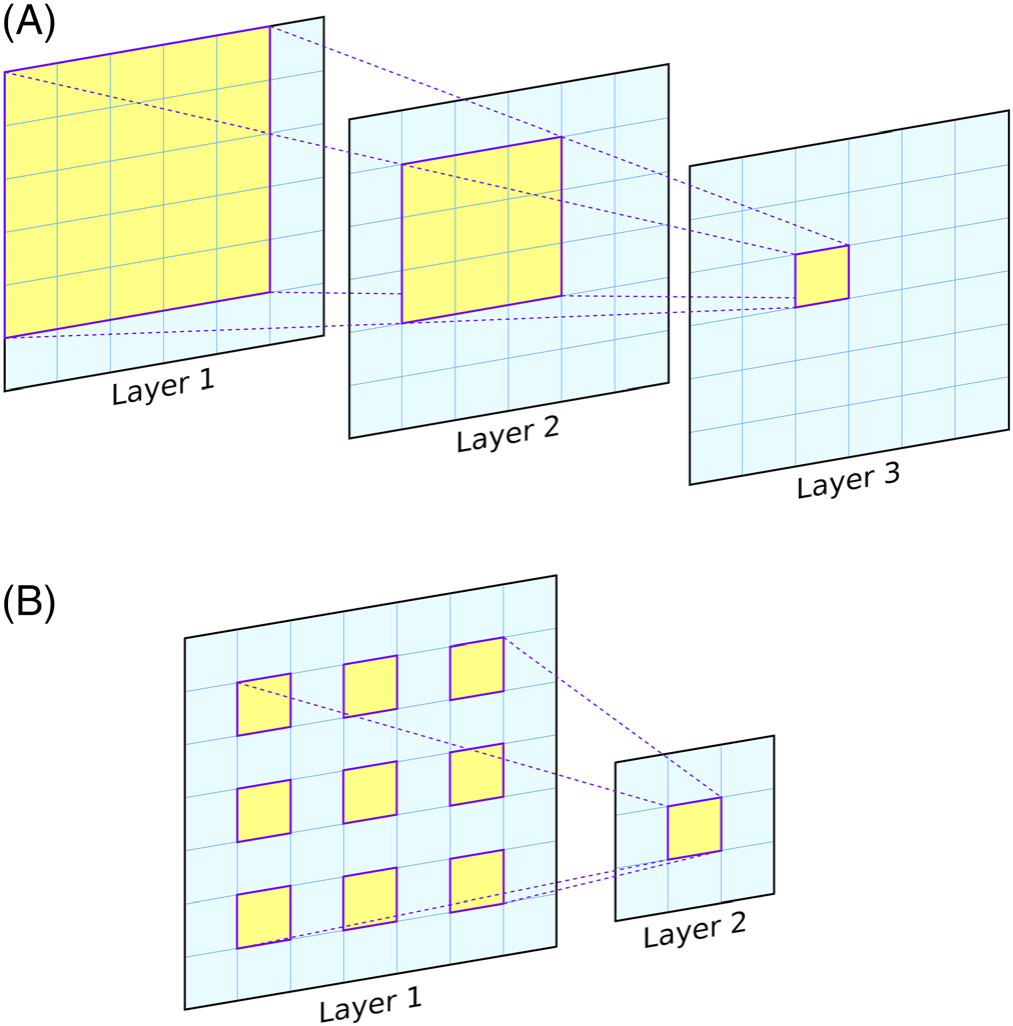
A, Illustration of the growth of the receptive field of a 2D CNN as convolutional layers are added. The 6 × 6 grids represent the output from three consecutive convolutional layers with filter sizes of 3 × 3, and information flows from layer 1 to layer 3. A single output at layer 3 (yellow cell) is obtained using a 3 × 3 window of inputs from layer 2. Each of these nine cells in layer 2 uses a 3 × 3 window of values from layer 1. These windows overlap, and the set union of the cells used by the highlighted cells in layer 2 is marked on layer 1 (5 × 5 grid). Thus, from the point of view of each output cell in layer 3, the receptive field is 5 × 5 cells in layer 1. B, A single dilated convolutional filter is shown, with a 3 × 3 filter and a dilation rate of 2. This layer has a receptive field of 5 × 5 despite having only nine adjustable weights. Stacking dilated convolutional layers allows the receptive field to grow exponentially using a linearly increasing number of parameters. In contrast, both the receptive field and the number of adjustable parameters grow linearly when using regular convolutional layers, as shown in A

An important parameter of CNNs is the *receptive field*. This simply refers to the area of the input image (or more generally, the input feature set) that can be “seen” at any one time. Concretely, the receptive field is the spatial extent of the inputs that are used in the calculation of a single output value, and is typically calculated for a single neuron in a given convolutional layer in the network (most commonly the last). Output neurons in a network comprising a single layer of 3 × 3 filters would have a 3 × 3 receptive field, as the final calculation carried out by the network for each output pixel only considers a central pixel and its immediate neighbors in the input (Figure 1). Composing a model with successive convolutional layers, however, can grow the receptive field, that is, the area around each input pixel that can be included in calculating an output in the final layer (see Figure 2A). A caveat is that the size of the receptive field is bounded by the size of the input; a CNN can be configured to have a large receptive field by adding more convolutional layers, but if it only ever operates on inputs with spatial dimensions of, say 32 × 32, then the receptive field can only grow to a maximum size of 32 × 32, regardless of the number of layers, even though its “theoretical” receptive field may be much larger. In practice, the maximum receptive field needs to be large enough to capture the relevant structures in the input data. Dilated convolutions^40^ can also be used to increase the receptive field with far fewer layers. In a dilated convolution, each filter is “stretched” by including spaces between each pixel (Figure 2B). A 3 × 3 filter with a dilation rate of 2 would actually cover the same area as a 5 × 5 filter, but with only nine learnable parameters rather than 25 (Figure 2B). The downside would be that the dilated filter will only be able to sample nine out of the 25 pixels and so will have “gaps.” However, these gaps can be filled by later dilated layers, so a network built with a mixture of dilated filters can cover an arbitrarily large receptive field without requiring an exponentially growing number of learnable parameters. In CASP13, dilated convolutions were used in a number of the top‐performing CNN models.^7^, ^41^, ^42^

Typical CNN models (eg, for image classification) take the output of one or more convolutional layers and usually downscale them with a “max pooling” operation. Max pooling simply looks for the maximum value within an area, but this operation reduces the size/resolution of the image. Ultimately, the final max pooling output is used as input to one or more fully connected layers. The output (using a softmax function usually) of the last fully connected layer will represent the output of the network, which is typically a classification of the input image into a fixed number of predefined categories (“cat” or “tree” for example). It is, however, also possible to have CNN models that take in image‐like inputs and produce image‐like outputs. This is achieved using fully convolutional networks (FCNs; not to be confused with fully connected networks),^43^ which are simply composed of a stack of convolutional layers all the way up to the output, omitting max pooling or fully connected layers that either change the image resolution or lose the image structure. Thus, an attractive property of FCNs is that they can be configured to produce output images of the exact same dimensions as the input. An example application of such a setup is to take in an image and produce an identically sized output image that highlights particular objects in the input image, which is known as image segmentation. In structural bioinformatics, this type of architecture has been used to great effect in contact prediction by a number of groups,^42^, ^44^, ^45^, ^46^, ^47^, ^48^ where the inputs to the network are one or more features dependent on the (squared) length of the target sequence (eg, amino acid covariance matrices), and produce outputs (contact maps) of the same shape.

**Figure 3:**
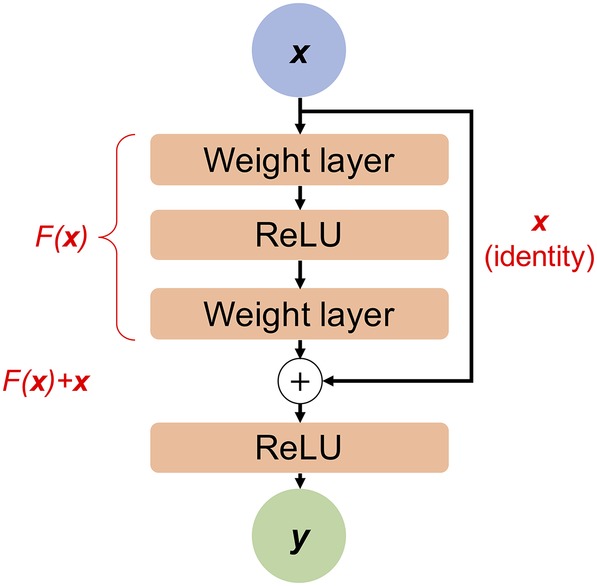
Illustration of data flow in a residual block. Red text illustrates the overall mathematical form of the transformations achieved at various steps. Input data ***x*** enters the residual block at the top, and flows through a number of weight layers (such as fully connected or convolutional layers), and usually includes one or more nonlinear activations (in this case, ReLU). The whole transformation up to this point is represented by *F(**x**)*. The result of these weight layers is then added to the input ***x***, before passing through another nonlinear activation layer. Other “wirings” of residual blocks are possible and have been studied.^49^ ReLU, rectified linear units

As already mentioned, training very deep CNNs can be difficult due to vanishing gradients. Although the solutions already discussed allow fairly deep nets to be trained (up to say 20 layers), for really deep networks, the gradients still end up getting lost and so such nets are very hard to train. To train very deep CNNs, therefore, a further trick has proven useful. To train a very deep FCN, skip‐connections or short‐cuts can be used that can bypass some layers and provide information from earlier layers directly to later layers^25^ (see Figure 3). These so‐called residual NNs (ResNets) are becoming the standard architecture for training very deep CNNs, and have been used in the best‐performing methods for contact and distance prediction in CASP,^9^, ^10^, ^41^, ^42^, ^47^, ^48^ including those by the Zhang and A7D groups in CASP13.

As we briefly mentioned earlier, CNNs are not just limited to 2D problems. Also of use recently have been 3D CNNs, where the input is generally a representation of the protein tertiary structure and the output is a measure such as an estimation of model accuracy^50^ or a binding site prediction.^51^ In this case the filters learn favorable residue orientation patterns, analogous to energy calculations using force fields in molecular mechanics. Although 3D CNNs often have large memory requirements, recent technical developments in this area indicate that it could well increase in importance in future CASP experiments.^52^

### 3.1 CNNs in contact prediction

In contact prediction, CNNs have proven themselves to be significantly more effective at the problem than the global statistical models that created a lot of excitement in the field just a few years ago. Examples of such global models include direct‐coupling analysis (DCA),^53^, ^54^, ^55^ pseudolikelihood maximization,^56^, ^57^, ^58^, ^59^, ^60^ and sparse inverse covariance estimation (SICE).^61^ As mentioned previously, given the correspondence between residue covariance matrices and contact maps, it is natural to treat these as image‐like inputs in order to derive a mapping. CNNs are ideally suited to such prediction problems, as the key idea in convolutional layers is to recognize local patterns regardless of their spatial position in the input. Taking this idea into the realm of contact prediction, applying convolutional filters to an amino acid covariance matrix, say, allows the model to detect interactions between local sequence motifs that are separated by an arbitrary number of residues,^45^ which corresponds nicely to observed structural patterns (eg, variable length loops or even entire domain insertions can be accommodated with no changes needed to the model).

The fact that CNN models, in which the key functional units are designed to only use local subsets of the data, can outperform global models in which all residue covariation data are considered simultaneously, can at first glance seem surprising. On the other hand, the ability to stack successive convolutional layers to increase the overall receptive field of the model theoretically allows the model to use as much of the covariation data for a target protein as necessary when predicting individual contacts. In a recent work,^45^ we created CNN models with varying sized receptive fields in order to assess whether a completely global view of the covariation data is necessary in order to achieve high precision when predicting contacts. We found that increasing the receptive field of the network led to increased precision, as might be expected, but significant gains were realized only up to a maximum receptive field size of 15 residues or so; further increases in the receptive field size (up to the evaluated maximum of 49) led to little or no gain in mean precision. The model, which we termed DeepCov, was also found to be significantly more precise than the global statistical model predictor, CCMpred,^59^ and equaled or outperformed our best available method at the time, MetaPSICOV2.^39^ These results suggested that most of the information needed to accurately predict contacts between a pair of residues lies in the residue covariation data for seven or so residues on either side of the residue pair being considered. The distance prediction model used by the A7D group in CASP13^10^ is a deep convolutional ResNet that uses 64 × 64 residue crops of the input features, and so by definition has a maximum 64 × 64 receptive field despite using a very large number of layers. The team was clearly able to produce highly accurate models using the predictions from this restricted model. However, the fact that the A7D team used a global statistical model (pseudolikelihood coupling parameters) to generate their input features makes the receptive field interpretation far less clear than for DeepCov, as predictions of distances for any residue pair could in theory depend on residue covariation statistics for the entire protein.

## 4 WHY IS DEEP LEARNING EFFECTIVE?

We now give some personal thoughts as to why we think DNN‐based models are effective at various problems, as well as some potential pitfalls.

### 4.1 Learning hierarchical representations

As each successive layer in a DNN composes the outputs from the previous layer, features of the data can be learned at varying levels of abstraction, allowing deeper models to recognize increasingly complex patterns. Taking as an example the task of recognizing faces in images, early convolutional layers can learn very simple features of the image, such as edges. Subsequent convolutional layers can then compose edges and learn to detect simple shapes, individual facial features, and finally whole faces. It is possible to inspect the convolutional filters in a trained CNN, and although these levels of abstraction are not always arranged neatly across successive convolutional layers, a hierarchy of features can often clearly be seen.^62^ Such an ability to learn hierarchical features can be useful in a variety of structural bioinformatics tasks, as information in biological data often does exist at various levels of hierarchical organization. For example, in proteins, information exists at the levels of individual residues, sequence motifs, fragments, secondary and supersecondary structures, domains, and so on. In the realm of contact prediction, Liu et al^63^ show that an FCN trained to predict interresidue contacts can recognize specific contact patterns between elements of secondary structure, although the work only inspected the patterns learned in the first convolutional layer in a simplified version of the model.

In general, DNN models work well on data that exhibit structure such that some form of hierarchical *parsing* is both possible and meaningful. For this reason, DNN models are generally not particularly effective in modeling unstructured data, which is unfortunately very common in many areas of biology and medicine. By unstructured data, we mean data where there is no geometric relationship between the inputs for example, features such as “cost,” “height,” “molecular weight,” “radius of gyration,” and so forth. In bioinformatics, an illustrative example of this is the DeepBipolar method^64^ that used CNNs to tackle the Bipolar exome task at the fourth Critical Assessment of Genome Interpretation (CAGI) experiment. Despite using a complex CNN architecture, it performed only slightly better than traditional classifiers such as random forests, likely due to the unstructured nature of the inputs (presence or absence of particular gene variant calls), but also, to be fair, possibly due to the limited amount of available training data. One useful take‐home message we can take from this is that deep learning has only proven to be effective in a fairly narrow (but still important) range of problems, and is not going to be the best approach in every machine learning application area.

### 4.2 Deep learning as a neighborhood density estimation method

In order to find better ways of using deep learning in future CASP experiments, it is valuable to have in mind a high‐level concept of what it is that these networks are actually doing. One way to think of what a NN actually does is that it acts as a highly sophisticated lookup table. During training, the NN places the features of each piece of training data in a high‐dimensional space. The shape of this space is learned alongside the data, but ultimately the end result is not that complicated. Given a particular set of inputs, the trained network computes the similarities between these particular inputs and representations of all of the inputs it has ever seen before in training. If the network is very large, and the amount of training data relatively small, then the network really does ultimately become a trivial lookup table. In that scenario, the training data are effectively stored within the weights of the network and during inference the network simply assigns a weight to these stored patterns and produces as output a weighted average of the original training outputs. A network like this is essentially useless for making predictions because unless the input is really close to one of the training examples, it will simply produce a output an average of all of the original training outputs, which is unlikely to be informative. This is what we mean by overfitting in the context of NN training.

It is easy to see that an overtrained model would exhibit very low to zero training error and could be obtained by a relatively straightforward optimization of a loss function. A number of *regularization* techniques can be used to avoid overfitting, such as adding penalty terms to the loss function (eg, L1 or L2 penalties, which are commonly used in regression models^65^, ^66^, ^67^), Dropout,^68^, ^69^ or early stopping,^70^ to name a few. Besides regularization, the main key to avoiding overfitting is always to collect a lot more data, but some benefit can be gained from simply reducing the complexity of the network. Reducing the complexity of the network basically means reducing the number of adjustable weights or parameters, and so typically means either using fewer layers, and so producing a shallower network, or making each layer narrower that is, reducing the number of weights in each layer. This model complexity reduction forces the NN to be more “creative” in the way it stores the training input–output pairings. It still tries to memorize the training data, but because it no longer has enough memory capacity to simply store the information, it is forced to make use of more complex representations of the data to store the same amount of data in less memory. This of course is recognizable as data compression, and so another useful way of looking at a NN is that it is learning how to compress the input training data. The better this compression is, the more we assume the network has learned about the underlying shape or structure of the data. Of course there must be limits to this, otherwise we would opt to use a NN with just a single adjustable weight to learn any sized data set. As with any compression method, data can only be compressed so much until it is simply not possible to reconstruct the original input to any kind of useful accuracy. This is what we refer to as underfitting a NN model.

Having illustrated the dangers of over‐ and underfitting, it is worth pointing out that most DNNs used in practice are overparameterized, that is, they have far more adjustable parameters than they have examples in the training set. Despite this, many such models can successfully generalize to new data, indicating that they are not overfitted to their training sets. Empirically, it has been found that overparameterization has the desirable side‐effect of producing models that are easier to train,^71^, ^72^ meaning that once the gradient‐based weight optimization process has reached a stationary point, the resulting model is found to be highly performant. Getting high performance from an overparameterized model (while avoiding overfitting) is possible due to the creation of an easier optimization landscape to be solved during the gradient‐based training process.^73^, ^74^, ^75^ Thus, somewhat paradoxically, overparameterized models do often show better generalization than underparameterized models. There is a tension between keeping a model parsimonious while on the other hand having sufficient complexity to model the data usefully, and crucially, so that the model can be trained effectively. As always, the pragmatic approach is to test various model architectures for a given problem and use the best one as determined from a held‐out test set.

Turning once again to CASP, the previously outlined developments in using deep convolutional NNs to predict either interresidue contacts or more usefully interresidue distance distributions has clearly had an impact, and there appears to have been an advance in our general ability to model protein structures without reference to a template structure. But is this really true? Have these DNNs looked through all of the structures in the protein data bank (PDB) and simply learned how proteins fold from first principles? If so, can a complete solution to the protein folding problem be just a few short years away from realization? That cannot be absolutely ruled out, but the likely answer to both these questions is probably no. To see why this is, we have to look at the basis of these exciting developments in protein structure and to place them in the context of what has happened in the previous 25 years or so of the CASP experiment. In many respects, deep learning‐based prediction of contacts and distances has superficial similarities to fold recognition and fragment‐assembly methods, in that they all make predictions by referencing some parts or all of a set of known structures.

Considering the NN models used in A7D (DeepMind's AlphaFold),^10^ RaptorX,^8^ or our own DMPfold,^9^ it is important to understand the limitations of what these models are capable of doing. The first thing we notice about these models is that they employ a variety of sequence‐derived input features. The majority of these features encode evolutionary information of various kinds, particularly direct coupling features, which have also had an impact on the last few CASP experiments. Indeed, DCA and SICE predictions have on their own been used successfully to fold proteins.^53^, ^60^, ^76^, ^77^, ^78^, ^79^ It should therefore come as little surprise that models that take these features as input are successful at predicting contacts, as in these cases the model does not have to achieve much more than “clean up” the initial predictions by recognizing local contact patterns across the training set. However, some DNN‐based contact prediction methods do not use direct coupling features, and instead operate on raw residue covariance matrices^45^ or even the input multiple sequence alignment (MSA) directly.^80^

Regardless of whether direct couplings, covariances, or the MSA itself is used, these features are arguably a very long way away from the simplest case of encoding a single amino acid sequence as the sole input. This means that a network using these features is almost certainly not learning anything specifically about the actual target sequence, but instead is learning statistical features of the family to which the target sequence belongs. This immediately creates a limitation on what the network can ultimately learn. If the inputs comprise information summarized from hundreds or perhaps thousands of different proteins, then it is clearly not ideal to train the network to associate this input with just a single target set of distances. Each member of the family will have a slightly different structure from every other member, and so this creates an inherent accuracy limit to deep learning‐based modeling, at least in its current form. At best, the network can learn the structure of an “average” member of the family, but at worst the known structure used in training could turn out to be something of an outlier. In that case the network will likely model that family fairly inaccurately, as it has been provided with a highly biased sample of the ensemble of structures represented in the family. This bias will vary from protein family to protein family, but it is reasonable to guess that the average of these biases is going to be somewhere in the region of 3‐5 Å RMSD. So, without many more samples of known structures, which would require a huge increase in the number of structures in PDB, covariation‐based deep learning models will likely only ever directly produce predictions of around this accuracy. This perhaps emphasizes the increased importance of structure refinement to the future of protein structure prediction, as “fold level” modeling may well effectively become a solved problem within a few years.

### 4.3 Robustness to missing or noisy inputs

An aspect of DNN‐based predictors that can be surprising is that they seem able to produce reasonable predictions even when the inputs are noisy or sparse (incomplete). An example in contact prediction is when the input MSA only has very few sequences in it.^45^, ^47^ With very few sequences, DCA‐ or other global model‐based predictors struggle to predict contacts, as there is too little information with which to fit the parameters of the global model accurately. Why then, can a DNN seemingly conjure up a reasonable contact prediction where other predictors cannot?

An accurate and deep MSA generally provides a reliable estimate of residue covariation along the entire protein chain. Conversely, shallow alignments comprised of only a few sequences or sequence clusters provide noisy and less reliable estimates of residue covariation. Low sequence diversity in the MSA is a particular problem: Pairs of columns in an MSA showing no variation at all will be assigned a covariance value of zero, and the presence of a large number of such pairs in the MSA essentially means that one has large amounts of missing data. This is a problem for the global statistical models as the calculations are performed using probability estimates for every amino acid type at every pair of sequence positions. Pseudocounts are generally used to “fill‐in” data for unobserved pairs, and for deep alignments we would expect that most of the parameters used in fitting come from actual observations rather than pseudocount guessing. For shallow alignments, however, *most* of the parameters will have been guessed rather than observed. So, it should not be at all surprising why global statistical models fail completely when given only shallow alignments.

**Figure 4:**
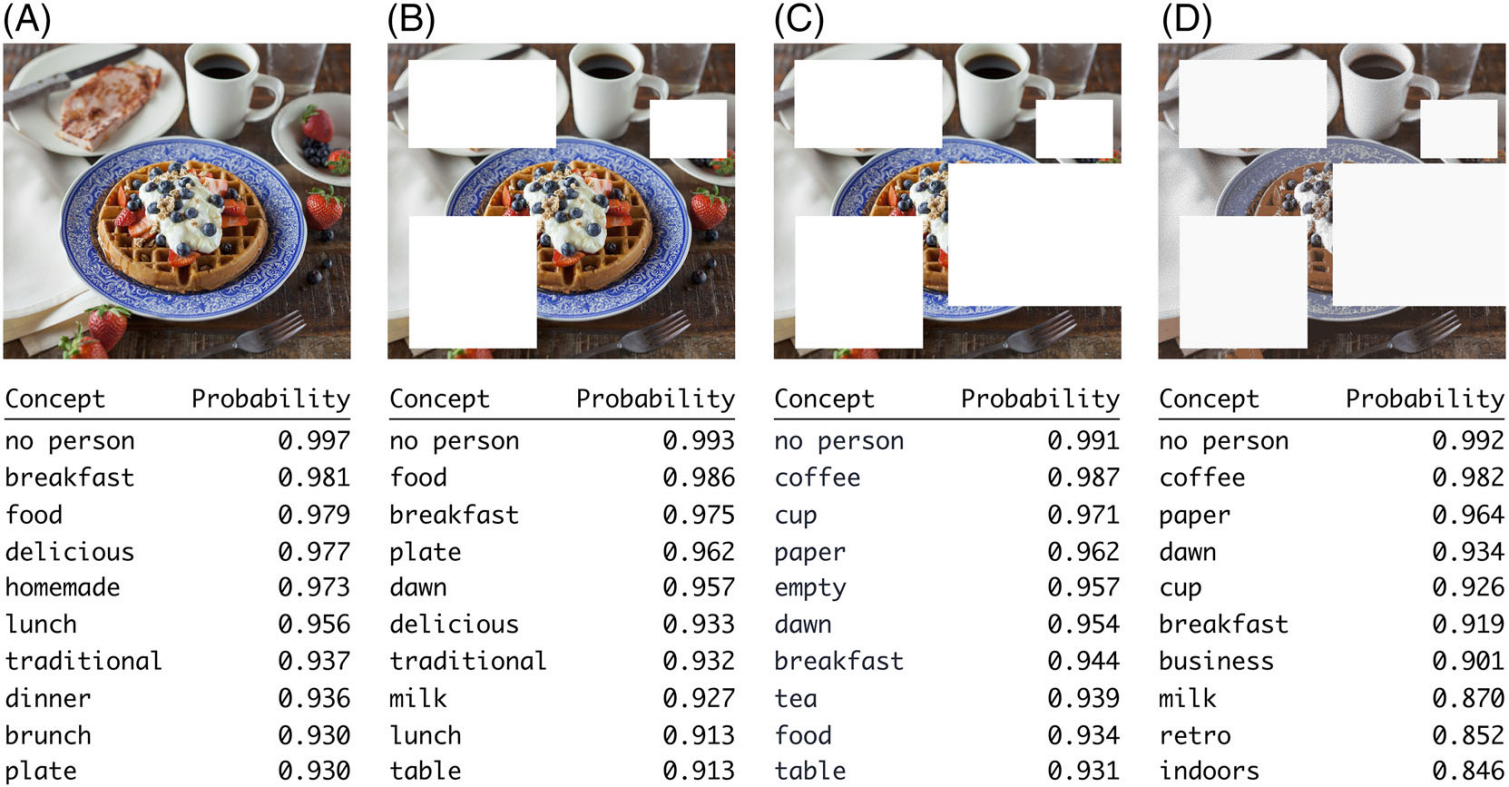
Well‐trained DNN models can be robust to missing or noisy data. Four versions of a sample input image are shown. Beneath each image, the top 10 predictions from an image classification model are shown. Panel A shows results for the original image; panels B and C show results for versions of A with data progressively “censored”; and panel D shows results for the same image as in C but using only eight colors. Although the predictions get worse going from panels A to D, there are still a few reasonable predictions in the top 10. The model used is the “general” image classification model available at: https://clarifai.com/demo. *Source*: Joseph Gonzalez on Unsplash

In contrast, when properly trained, DNN models can be quite robust to missing data. Figure 4 illustrates this idea using an image classification model that is available online. Given an input image, this model is trained to predict scores for a predefined set of concepts that describe the content of the image. Figure 4A shows the 10 highest‐scoring predictions for the original image. In Figure 4B,C, a significant fraction of the image has been “censored” or more technically “ablated,” mimicking a situation where one has missing data. The model clearly still returns reasonable predictions, although the overall accuracy is clearly reduced as more data get ablated. If we consider the task of contact prediction from just raw covariance data, when an input MSA has only a few sequences, the covariance estimates can only take on a limited set of values. In the realm of digital images, this is (very loosely) similar to using only a limited number of colors for an image. Once again, properly trained DNN models for image recognition can be surprisingly robust to this effect (Figure 4D).

Robustness to missing or noisy data is not necessarily a new aspect of deep learning (DL) models; instead, the lack of such robustness can be considered an obvious failure mode of global models such as DCA, pseudolikelihood methods, and SICE. When the inputs to such models are sparse, one is forced to use techniques such as shrinking the covariance matrix or adding pseudocounts so that the model has complete information to work with. DL models can simply be trained to naturally deal with such missing data without needing it to be explicitly filled in.

To better understand this, imagine an MSA with two distinct regions (this could be a two domain protein). One region has many aligned sequences, and few long gaps, the other region has few aligned amino acids and very many long gaps. An unsupervised covariation method such as PSICOV will struggle to produce an accurate contact map in this situation. This is because every decoupling equation in the calculation will include pairwise covariance terms from both the good and bad parts of the alignment. Thus, the whole predicted map will be of low accuracy because of the missing data in the bad section. A convolutional network method such as DeepCov, on the other hand, will have no such problems. The learned filters in the convolutional layers will still detect local patterns corresponding to real contacts in the good parts of the alignment, but will be unhindered by the noisy data from the bad part of the alignment. In the bad part of the alignment, the filters will simply detect few or no contacts. This is analogous to the way that convolutional nets outperform fully connected nets in image labelling tasks (see Figure 4, wherein a confident prediction for “breakfast” is still returned after much of the input image has been ablated). In the case of shallow alignments with more or less uniform coverage across the whole query sequence, the network would simply look at the data for those columns that do show some covariation, and possibly detect contacts in these regions. Additional benefits may be gained from the fact that the network has seen multiple proteins and MSAs during training, which is in contrast to the way in which global statistical models operate.

In fact, a convolutional network can be made even more robust by *augmentation* of the training data. In the case of contact prediction, this could involve showing the network multiple versions of input features for each target protein, where each version of the input is composed from MSAs of varying quality. An approach of this type has already been used to try to improve robustness in contact prediction.^42^

### 4.4 Potential pitfalls

Compared to application areas such as text or images, bioinformatics databases typically have far fewer data points that can be used to train predictive models. As DNN models frequently have millions of adjustable parameters, for sufficiently small numbers of training examples it becomes possible for DNNs to be easily overtrained and to memorize a training set, as we have described above. Perhaps the biggest problem with overtrained models is that we may overestimate their performance before they are applied to new problem instances. It is thus important to evaluate a model's ability to generalize to new, unseen examples, and to confirm that the model has indeed learned features of the input data that are predictive of the desired property(ies), rather than just memorizing the training set. One approach is to use a test set that has no overlap to the training set (but which obviously sits within the same problem domain that the model was trained on). The procedure chosen for defining the nonoverlap between the training and test sets is thus of crucial importance. In structural bioinformatics, the best procedure varies according to the prediction task. Considering DNNs that predict interatomic contacts or distances, for example, it is desirable that the model can accurately predict these properties for entirely novel folds. The only way to evaluate this reliably is to ensure that there are no proteins in the test set(s) used for benchmarking that have a similar 3D structure or fold as any protein in the training set. Structural classification databases such as CATH^81^, ^82^ and ECOD^83^, ^84^, ^85^ can be used for this very purpose, as some studies have wisely done.^45^, ^80^ As an example, eliminating training examples in the same CATH or ECOD T‐group as any testing example provides a satisfactory means to eliminate structural overlap between the two sets. Many papers, on the other hand, use a data set split based only on a sequence identity threshold, most commonly 25%‐30% sequence identity. While such a split may be sufficient in cases where structural information is not relevant, the problem is that sequences in the same family and with similar structures can have sequence identity well below 30%, and sometimes with 0% sequence identity. In this case, a correct prediction by the network is likely to be repeating what it has already seen during training rather than making a true prediction of the task at hand. This problem affects prediction tasks including secondary structure prediction and contact or distance prediction, to name but a few. We can think of vanishingly few problems for which a split based on sequence identity is sufficient; one example is function prediction, in which two highly sequence‐similar proteins can have very different functions. While it is difficult to encourage researchers to adopt a more stringent procedure that is both harder to implement and makes their benchmarking results look worse, a shift to using rigorous training and testing splits is essential if we are to accurately assess the impact of deep learning‐based methods, and for practitioners to trust the predictions provided by such models in the future.

## 5 CONCLUSIONS AND OUTLOOK

Research in NNs and deep learning continues to develop at a rapid pace, with ideas for new architectures, training tricks, weight optimization algorithms and other tools appearing on a weekly and sometimes daily basis. Indeed, there are a number of very important ideas that we have not covered in this article. Perhaps the most important is that of recurrent architectures, which map sequences of data to other sequences. Recurrent NNs are widely used in the prediction of secondary structure, solvent accessibility, disorder and backbone torsion angles.^86^, ^87^, ^88^ Recurrent architectures have also been used in contact prediction.^48^, ^89^ More recently, a recurrent architecture has been used to model tertiary structure.^90^ This latter method has the attractive property of being end‐to‐end differentiable, meaning that all parts of the process from taking in the input features to predicting 3D coordinates (via predicted torsion angles) can be simultaneously optimized during the NN training process. Other methods such as deep reinforcement learning^91^ and generative models (such as generative adversarial networks^92^ and variational autoencoders^93^) have not yet had a clear impact in CASP, but perhaps will in the future.

Deep learning is clearly taking bioinformatics by storm. As reviewed here, this is due to the ability of deep learning models to take into account different levels of structure in data, to deal with noisy data, to take in raw features without the need for feature engineering, and to interpolate sensibly to make reasonable predictions for data not used in training. This trend looks likely to continue for at least a few years due to a few reasons: constant improvements in hardware, architectures and algorithms; the ever‐increasing amount of experimental data collected; and the increasing crossover between the machine learning and bioinformatics communities. As deep learning for bioinformatics moves into a more mature phase it is essential that rigorous benchmarking and evaluation becomes more common in published literature. Of course, the ultimate aim of bioinformatics is not just accuracy on prediction tasks but understanding of the underlying biological processes at work. As research into the interpretability of NNs improves, it would be beneficial for successful networks in bioinformatics to be interrogated to see which features and signals are important. Such understanding could even be used to help the networks themselves become more robust and accurate.
